# Echocardiographic evaluation of thalassemia intermedia patients in Duhok, Iraq

**DOI:** 10.1186/1471-2261-14-183

**Published:** 2014-12-11

**Authors:** Ameen Mosa Mohammad

**Affiliations:** Department of Medicine, Division of Cardiology, Medical School, Faculty of Medical Sciences, Duhok University, Kurdistan, Iraq

**Keywords:** Thalassemia intermedia, Tricuspid velocity jet, Pulmonary hypertension, Iraq

## Abstract

**Background:**

Cardiac complications are among the most serious problems of thalassemia intermedia patients. The current study was initiated to address the latter issue through the study of the echocardiographic findings and correlate it with clinical characteristics of thalassemia intermedia patients in Duhok, Kurdistan region, Iraq.

**Methods:**

An echocardiographic assessment of 61beta-thalassemia intermedia cases was performed. It included 30 males and 31 females, with a mean age 19.6 ± 7.5 years. The standard echostudy of two-dimension and M-mode measurements of cardiac chambers were done. The continuous doppler regurgitant jet of tricuspid and pulmonary valves were recorded. Left ventricle diastolic function was assessed by pulsed doppler of mitral valve inflow. To correlate the clinical with echocardiographic findings, patients were divided, according to tricuspid regurgitant velocity, into three groups (<2.5 m/sec, 2.5-2.9 m/sec and ≥3 m/sec).

**Results:**

Tricuspid regurgitant velocity <2.5 m/sec, 2.5-2.9 m/sec and ≥3 m/sec occurred in 42(69%), 11(18%) and 8(13%) respectively. Comparing to other groups patients with tricuspid regurgitant velocity ≥3 m/sec were older and included more males. They had lower hemoglobin levels, but higher ferritin levels. Their age at diagnosis and the age of the initiation of blood transfusion were later. Most of them had significant exertional dyspnea. They also had relatively lower left ventricle ejection fraction values. Right ventricular diameter and right atrial size were larger in the same group. Tricuspid regurgitant velocity as a continuous predictor was associated positively with age, cardiac volumes and pulmonary regurgitation though negatively associated with ejection fraction.

**Conclusions:**

Echo-derived right and left side cardiac complications are not uncommon in thalassemia intermedia patients. Therapeutic trails targeting these complications are indicated, and echocardiographic assessment is necessary to be offered early for thalassemia intermedia.

## Background

Thalassemia Intermedia (TI) is an inherited hemoglobin disorder that is characterized by a significant genetic and clinical heterogeneity.

Cardiovascular involvement represents a well-known complication and the primary cause of mortality in TI. It is mainly determined by the fact that both ventricles have to maintain a high cardiac output level through a stiff vascular bed. Pulmonary hypertension and steady increase in tricuspid regurgitant jet followed by right heart failure dominates the clinical picture.

The left ventricle has to maintain a high cardiac output in a continuous state of both volume and pressure overload which consequently contributes to left ventricular impairment with time [1–3].

Several factors have been reported to interfere in the pathophysiology of cardiovascular abnormalities in TI. However, the key mechanism is the chronic tissue hypoxia and chronic hemolysis with its consequences [3]. The screening of patients with thalassemia intermedia for evidence of cardiac complications by transthoracic echostudy is a good option for early diagnosis and eventually for future planning of best management of cardiac complications. The purpose of this study is to look for evidence of cardiac involvement by echocardiography and correlate the echocardiographic findings with clinical characteristics of patients with TI.

## Methods

This is a prospective study of 61 beta-thalassemia intermedia patients (out of total 74 registered in Duhok thalassemia center). It included 30 males and 31 females, with a mean age of 19.6(SD7.5) years. All patients were evaluated by history taking and physical examination, laboratory studies, and underwent transthoracic echocardiography between June 2010 and June 2011. Either patients or their relatives provided written informed consent.

Transthoracic echocardiography was performed for all patients by Vivid 3(GE) Echo systems. The standard two-dimension and M-mode measurements of the cardiac chambers including left ventricle ejection fraction (LVEF %) were taken. Cardiac volumes and dimensions were indexed by body surface area. Tricuspid regurgitation was assessed in the apical four chambers, parasternal short axis views, and a minimum of three sequential complexes were recorded. Continuous doppler sampling of the peak regurgitant jet to estimate the right ventricular to right atrial systolic pressure gradient by the modified Bernoulli equation was obtained. To assess the mean pulmonary artery pressure, the pulmonary artery to right ventricular pressure gradient was estimated from the peak pulmonary regurgitant flow velocity (V) in parasternal short axis view by the simplified Bernoulli equation (PG = 4 V2) [4]. Left ventricle diastolic function was assessed by pulsed doppler sampling of mitral valve inflow in four apical chambers view. The peak E and A waves velocities and the E/A ratio of the mitral valve were recorded. All echocardiographic measurements were performed according to American society of echocardiography recommendations [5].

To correlate between the clinical and Echo findings within the studied sample, the patients were divided according to tricuspid regurgitant velocity (TRV) into three groups (TRV < 2.5 m/sec, 2.5-2.9 m/sec and ≥3 m/sec) [6]. Additionally, TRV was taken as a continuous variable to assess its associations with clinical and echocardiographic findings. Data of blood transfusion history were categorized into: regularly transfused (patients transfused at regular intervals every 1–3 months for last year prior to study enrollment) and irregularly transfused (≤ 3 units of blood/ year). Continuous variables were calculated as mean (SD) and categorical variables were presented as counts and percentages. ANOVA and a Fisher’s exact tests were used for the comparison of continuous and categorical variables respectively. Scatter plots with line regression were used for the analysis of TRV as a continuous variable with other predictors. P-value < 0.05 was considered significant. The study was approved by the ethical committee at the School of Medicine, Faculty of Medical Sciences, University of Duhok.

## Results

### Correlations of clinical characteristics to TRV

Since patients have been classified into three groups according to the TRV, 42(69%) had TRV <2.5 m/sec, 8(13%) had ≥ 3 m/sec, the remaining 11(18%) had between these two values. Patients with highest TRV were older. Almost all of them were over 12 years old. Their age at diagnosis and first blood transfusion were later. Majority of them were male with significant exertional dyspnea. Their Hemoglobin level was lower, but ferritin was higher. Most of them were splenectomized. BSA was higher in the same group as shown in Table 1.

**Table 1:** **Clinical characteristics of TI patients in relation to TR jet velocity**

Characteristics	Jet velocity <2.5 m/sec 42(69%)	Jet velocity 2.5-2.9 m/sec 11(18%)	Jet velocity ≥ 3.0 m/sec 8(13%)	p-value
Mean Age(year)	14.0(SD11.2)	19.0(SD 5.0)	26(SD 6.30)	0.007
Age ≥ 12 Years	19(45.23%)	8(72.72%)	8(100%)	0.004
Age at Diagnosis(year)	6.40(SD1.74)	8.02(SD1.06)	9.10(SD1.2 0)	<0.001
Sex(male)	16(38%)	8(72%)	6(75%)	0.01
BSA(m^2)^	0.98(SD0.37)	1.40(SD0.25)	1.46(SD0.13)	<0.001
BMI(kg/m^2^)	16.95(SD2.91)	18.09(SD2.15)	19.47(SD2.54)	0.05
Exertional dyspnea	4(9.5%)	3(27%)	6(75%)	<0.001
Hemoglobin level(g/dl)	9.10(SD1.2)	8.02(SD1.0)	7.90(SD1.10)	0.003
Ferritin level(ng/ml)	987.5(SD545)	932(SD674)	1650(SD824)	0.01
Age of 1^st^ BT^^^	7.50(SD5.70)	10.80(SD6.70)	13.7(SD6.20)	0.01
Regular BT^	11(23.8%)	2(9%)	2(12.5%)	0.8
Splenectomized	6(14%)	2(18%)	5(62%)	0.01

^BT = Blood transfusion. Data are presented as count (%) or mean ± SD.

### Echocardiographic findings in relation to TRV

Higher TRV values were significantly associated with increased cardiac chamber indices. These indices included left ventricle end diastolic diameter (LVEDD), left ventricle end systolic diameter (LVESD), left atrial (LA) diameter, and right ventricle (RV) diameter and right atrial (RA) size. There was a relatively slight decrease in the ejection fraction at the highest levels of jet velocity. There was an increase in the mean of LV wall and septal thickness with higher TRV values, though it did not reach the significant difference. The peak gradients of tricuspid and pulmonary valves were significantly associated with an increasing degree of TRV as shown in Table 2.

**Table 2:** **Echo findings of TI patients according to TR jet velocity**

Echo Findings	Jet velocity <2.5 m/sec 42(69%)	Jet velocity 2.5-2.9 m/sec 11(18%)	Jet velocity ≥3.0 m/sec 8(13%)	p-value
IVSDD(cm/m^2^)	0.51(SD0.20)	0.55(SD0.16)	0.56(SD0.13)	0.68
LVEDD(cm/m^2^)	2.90(SD0.51)	3.15(SD0.54)	3.40(SD0.40)	0.01
LVPWDD(cm/m^2^)	0.52(SD0.14)	0.53(SD0.15)	0.54(SD0.15)	0.92
IVSSD(cm/m^2^)	0.75(SD0.20)	0.76(SD0.21)	0.81(SD0.20)	0.74
LVESD(cm/m^2^)	1.56(SD0.35)	1.79(SD0.37)	1.91(SD0.40)	0.01
LVPWSD(cm/m^2^)	0.57(SD0.15)	0.59(SD0.13)	0.63(SD0.11)	0.54
LVEF (%)	65(SD6.40)	64(SD7.20)	56(SD12.40)	0.01
LA diameter(mm/m^2^)	22.53(SD5.0)	25.46(SD5.80)	27.25(SD6.60)	0.01
Transmitralinflow E/A ratio(m/s)	1.70(SD0.61)	1.42(SD0.40)	1.26(SD0.38)	0.07
RV diameter (cm/m^2^)	1.24(SD0.62)	1.57(SD0.71)	1.99(SD0.68)	0.01
RA area(cm^2^/m^2^)	9.11(SD4.20)	12.6(SD3.10)	13.52(SD3.40)	0.003
TAPSE(cm)	2.66(SD1.02)	2.33(SD0.93)	1.8(SD0.40)	0.06
TR peak gradient (mmHg)	21.55(SD10.23)	27.21(SD7.66)	35.44(SD9.1)	0.001
PR peak gradient (mmHg)	10.20(SD4.10)	13.25(SD6.20)	15.01(SD4.41)	0.01

EF: Ejection fraction, IVSDD: Interventricular septum diastolic diameter, LVEDD: Left ventricle end diastolic diameter, LVPWDD: Left ventricle posterior wall diastolic diameter, IVSSD: Interventricular septum systolic diameter, LVESD: Left ventricle end systolic diameter, LVPWSD: Left ventricle posterior wall systolic diameter, LA: Left atrium, RV: Right ventricle, RA: Right atrium, TAPSE: Tricuspid annular plane systolic excursion, TR: Tricuspid regurgitation, PR: Pulmonary regurgitation.

### Associations of TRV as a continuous variable with other predictors

Scatter plots of TRV with age and echocardiographic predictors showed a significant association with age, LVEDD, LVESD, LA diameter, RV diameter, RA area and PR peak gradient. But there was a negative association with LVEF % as shown in Figures 1, 2, 3, 4, 5, 6, 7 and 8.

**Figure 1:**
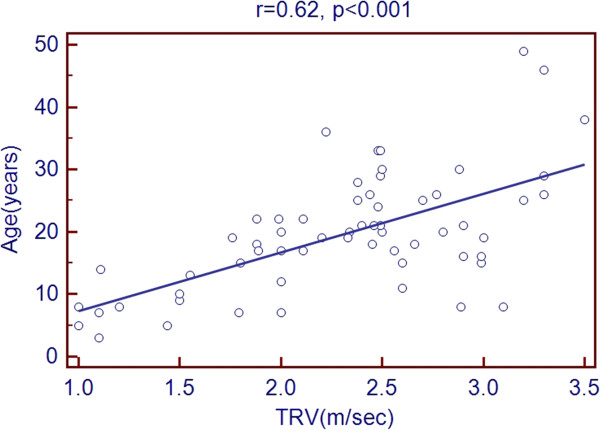
**Association of TRV with age of patients.**

**Figure 2:**
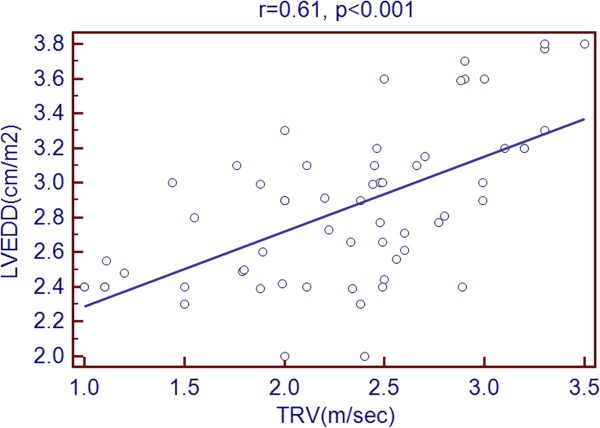
**Association of TRV with LVEDD.**

**Figure 3:**
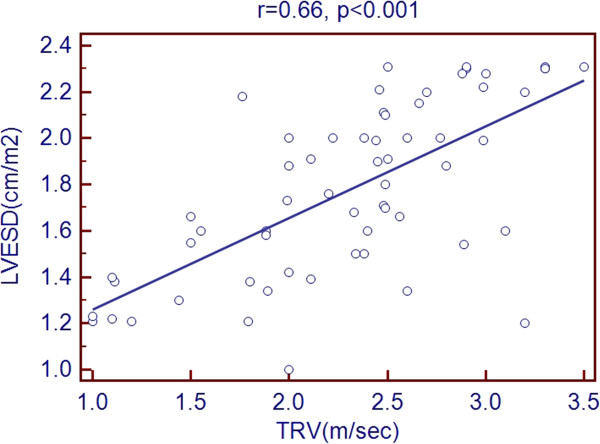
**Association of TRV with LVESD.**

**Figure 4:**
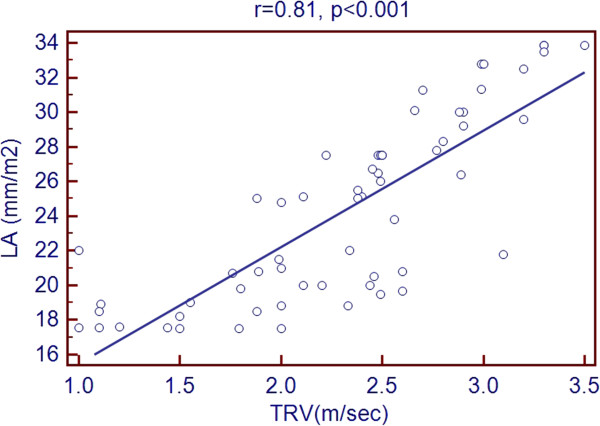
**Association of TRV with LA diameter.**

**Figure 5:**
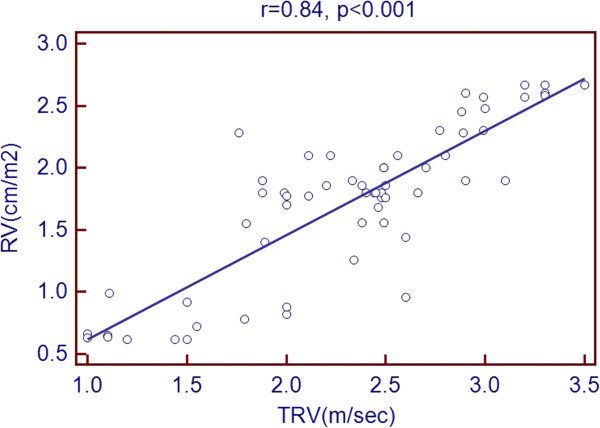
**Association of TRV with RV diameter.**

**Figure 6:**
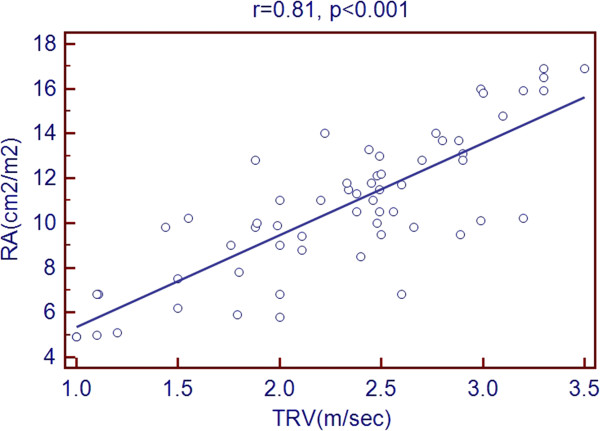
**Association of TRV with RA area.**

**Figure 7:**
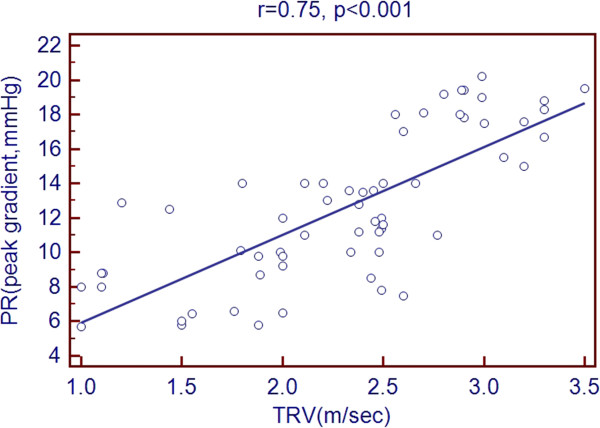
**Association of TRV with PR.**

**Figure 8:**
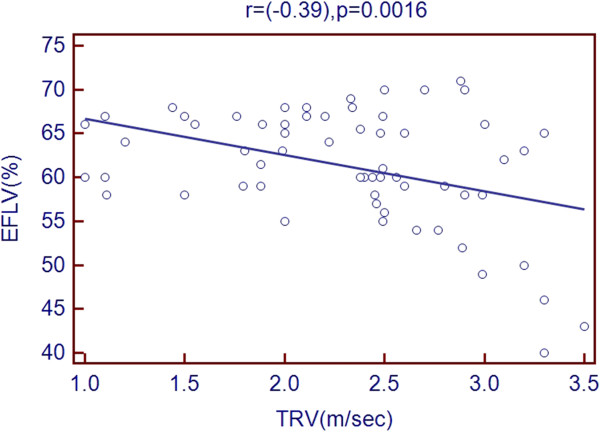
**Association of TRV with LVEF %.**

## Discussion

The main findings of the study are the following: first, both the left and right cardiac chambers, especially the dimensions and volumes, were increased in TI patients with time. Second, patients with higher TRV were presented with advancing age, later age at diagnosis, later onset of initiation of blood transfusion, predominant male gender, lower hemoglobin level, higher ferritin rates, and higher percentage of splenectomised patients. Third, the LVEF % was normal. It was relatively lower for the patients with higher TRV. Three patients had features of congestive heart failure.

Some of TI patients have an exceptional hemodynamic pattern consistent with right ventricular cardiomyopathy and PHT as well as the left ventricular abnormalities that can be a leading cause of cardiopulmonary problems in these patients. Frequent monitoring of cardiac function in patients may indicate that those at risk of developing symptomatic cardiac disease. These patients might then become candidates for more intensive therapy including blood transfusion and sustained iron chelation [7, 8].

The present study showed that the left ventricular dimensions including (LVEDD and LVESD) in TI were increased with an increase in the TRV. This is in consistence with previous studies by Nouri et al., Aessopos et al. and Vaccari et al. [9, 2, 10]. The mean of LV septal and posterior wall dimensions during systole and diastole were increased in spite of statistical insignificance. This finding is consistent with those of Aessopos et al. and Nurri et al. studies [2, 9]. The larger LV dimensions could be explained by the presence of chronic anemia, which is associated with increased blood volume. The lower capacity of blood to carry an adequate amount of oxygen to peripheral tissues was overcome by higher cardiac output and the venous return was, therefore; increased. This significant volume overload was carried out through the Frank-Starling mechanism [10].

Evaluating LV systolic function, the mean LVEF % in the studied sample was normal. There was a negative association between TRV and LVEF % with correlation coefficient equal to (-0.39). However, comparing LVEF % among subgroups, there was a significant difference because of a possible skew caused by a very small highest TRV sample that included three patients with features of congestive heart failure. According to Aessopos et al. [3] the LVEF % was normal in TI. However it was lower than LVEF % of control group with a significant difference (p < 0.05) [3]. Noori et al. [11] found significant lower values of LVEF % among TI compared to control group (p = 0.001). Vaccari et al. study showed lower LVEF % among TI though insignificant compared to control group [10]. Tissue doppler, a recently introduced method, is known to permit early identification of systolic dysfunction even when left ventricular is still preserved. In this study, however, it was not used [12–14].

Assessing LV diastolic function in TI patients, the mean LA volume and diameter were significantly higher in TI with higher TRV. There was also a progressive increase of the late filling velocity and a relative decrease of the mitral inflow E/A ratio though statistically insignificant. This suggests an increase in LV end diastolic pressure, reflecting an alteration in diastolic property and impairment of LV relaxation, most probably caused by iron deposition which gradually, if not properly managed, leads to restrictive cardiomyopathy [12, 15, 16]. The left atrial diameter in Aessopos et al. [3] was significantly larger in TI compared to control and the peak late transmitral diastolic velocity was significantly higher in TI. On the other hand the E/A ratio in Vaccari et al. and Noori et al. studies were significantly lower in TI compared to control group [10, 11].

Congestive heart failure (CHF) in this study was encountered in 3(4.9%) patients. Yet, the rates of CHF in Aessopos et al. [2] and Aessopos et al. [17] were 2.7% and 5.4% respectively. In TI, the heart is primarily affected by PHT, which is the leading cause of CHF due to subsequent right ventricle dysfunction [18]. The current study showed that there was a decrease in the value of tricuspid annular plane systolic excursion in higher TRV subgroup. However, it did not show a statistical significant difference.

In the present study 8(13%) of cases had TRV ≥ 3.0 m/sec which corresponds to doppler peak systolic tricuspid gradient (PTG) greater than 30 mm Hg and 6(9%) had TRV > 3.2 m/sec. Despite the lack of cardiac catheterization data the last value was considered to represent the actual cases of PHT in this study relying on diagnostic conclusions of PHT from a recent Italian study [19]. In Aessopos et al. (59.1%) had PTG greater than 30 mmHg and (23%) had PTG > 35 mmHg [17]. The observation that markers of hemolysis are associated with pulmonary hypertension in chronic hemolytic disorders suggests that there is a distinct syndrome of hemolysis-associated pulmonary hypertension. Following the release of hemoglobin into plasma, the plasma hemoglobin can scavenge nitric oxide as well as catalyze the formation of reactive oxygen and nitrogen species, the processes that can lead to acute and chronic pulmonary vasoconstriction [20]. With regard to the TI clinical characteristics, a retrospective analysis showed that splenectomised females with significant anemia, thrombocytosis and elevated ferritin levels, were at greatest risk for developing PHT [21, 22].

This study, similar to above analysis, proved that TRV was significantly higher in splenectomised cases, lower hemoglobin, higher ferritin levels, and later onset of blood transfusion. On the contrary, it concluded that higher TRV was associated with male gender. Unsurprisingly, despite of less regular blood transfusion among higher TRV subgroup in the year before this study, relative higher ferritin levels among them was found which was partly attributed to lesser utilization of iron chelation in the same subgroup, the increased risk of progressive ferritn accumulation with advancing age and increased rate of gasrointerstinal iron absorption in TI [23, 24].

A recent study demonstrated a significant correlation between iron overload in the liver and pulmonary artery systolic pressure independent of LV filling pressures [25]. Though this study had made no data with regard to iron overload in the liver, a significant association between higher ferritin levels and increase in pulmonary artery systolic pressure and higher jet velocity of tricuspid regurgitation was found. The use of serum ferritin to monitor iron overload is inexpensive and accessible. In TI, serum ferritin assays may underestimate the actual iron load and liver iron concentration (LIC) is more reliable, though invasive [26].

Managing TI patients with cardiac involvement and PHT, regular transfusion and iron chelation therapy is indicated even without evidence of cardiac siderosis. This suggests that TI patients may still be at risk for iron related cardiac dysfunction through exposure to non-transferrin bound iron [27, 28]. However, only 25% of the studied patients did receive regular blood transfusion, and even among patients with cardiac manifestations only few cases received iron chelation therapy. Sildenafil has been successfully used to treat PHT. However, none of the patients have had received the drug [29]. Hydroxycarbamide may be administered in TI patients in order to minimize, or even obviate, the need for regular transfusions and concomitant iron overload, and eventually minimize the cardiac involvement. Again, only a few cases have been receiving the drug, and with fair outcome and compliance [30].

The main limitations of this study, which is, to my best knowledge, the first attempt conducted in Iraq on echocardiographic evaluation of TI, include reliance on conventional echocardiography. Other diagnostic tools, such as tissue doppler, speckled tracking echocardiography, cardiac catheterization, and cardiac MRI, were not accessible. The studied sample is relatively small to generalize the outcomes, though it resulted in a correlation between accumulative changes in the clinical characteristics and echocardiographic parameters.

## Conclusions

Conventional echocardiography for TI cases is a useful tool for preliminary evaluation of cardiac status. It could uncover many cardiac abnormalities that possibly complicate TI. The abnormalities and the underlying pathophysiology could be targeted in the future.

## Authors’ information

The author is a senior cardiologist at Duhok heart center and teaches cardiology at Department of Medicine, University of Duhok, Iraqi Kurdistan.

## Abbreviations

BT: Blood transfusion
CHF: Congestive heart failure
EF: Ejection fraction
IVSDD: Interventricular septum diastolic diameter
LVEDD: Left ventricle end diastolic diameter
LVPWDD: Left ventricle posterior wall diastolic diameter
IVSSD: Interventricular septum systolic diameter
LVESD: Left ventricle end systolic diameter
LVPWSD: Left ventricle posterior wall systolic diameter
LA: Left atrium
PTG: Peak tricuspid gradient
RV: Right ventricle
RA: Right atrium
TAPSE: Tricuspid annular plane systolic excursion
TR: Tricuspid regurgitation
PR: Pulmonary regurgitation
PHT: Pulmonary hypertension
TRV: Tricuspid regurgitant velocity
TI: Thalassemia intermedia.
